# Draft genome sequence of non-pathogenic *Escherichia coli* 5C LMG S-33222, isolated from healthy donor feces

**DOI:** 10.1128/mra.00580-24

**Published:** 2024-09-16

**Authors:** Francesco Di Pierro, Nicola Zerbinati, Luigina Guasti, Massimiliano Cazzaniga, Alexander Bertuccioli, Chiara Maria Palazzi, Edoardo Labrini, Valeria Sagheddu, Sara Soldi

**Affiliations:** 1 Microbiota International Clinical Society, Torino, Italy; 2 Scientific & Research Department, Velleja Research, Milano, Italy; 3 Department of Medicine and Surgery, University of Insubria, Varese, Italy; 4 Department of Biomolecular Sciences, University of Urbino Carlo Bo, Urbino, Italy; 5 AAT-Advanced Analytical Technologies, Fiorenzuola d’Arda, Piacenza, Italy; Department of Biological Sciences, Wellesley College, Wellesley, Massachusetts, USA

**Keywords:** probiotics, proteobacteria, colibactin

## Abstract

We present the genome sequence of *Escherichia coli* 5C LMG S-33222, a non-pathogenic microorganism with potential probiotic features. The strain was isolated in 2021 from a fecal sample of a healthy Italian infant. The total genome size is 4,712,575 bp with a G + C content of 51%.

## ANNOUNCEMENT

Probiotic products predominantly consist of *Bifidobacterium*, *Lactobacillus*, and *Saccharomyces* (1), with some countries commercializing *Escherichia coli* as Colinfant New Born (CNB), Mutaflor, and Symbioflor (2, 3).

We present the draft genome sequence of *E. coli* 5C LMG S-33222, isolated from healthy Italian newborn feces (44°55′22″80 N, 09°54′36′00 E). The strain survives the simulated digestive process and adheres to enteric cell lines. Feces were serially diluted in Maximum Recovery Diluent plated on Tryptone Bile X-glucuronide agar medium (Merck, Milan, Italy) and incubated at 37°C. Informed written consent was signed in compliance with the Helsinki Declaration and the study was exempted by the ethical committee. DNA was isolated according to Chen et al. (4). The V1-V9 16S rRNA gene was amplified using P0 (27f) (5′-GAGAGTTTGATCCTGGCTCAG-3′) and P6 (1495r) (5′-CTACGGCAACCTTGTTACGA-3′) primers (5). The amplicon was sequenced by Sanger using SeqStudio Genetic Analyzer (BMR Genomics, Padua, Italy). The nucleotide sequence was deposited (GenBank PP951836), and aligned with nucleotide BLAST (v.2.15.0) finding CP158275.1 as the closest correspondence (6).

Antibiotic resistance profiles were evaluated using Clinical and Laboratory Standards Institute (CLSI) method and European Food Safety Authority (EFSA) guidelines, comparing minimum inhibitory concentration values and breakpoints for Enterobacteriaceae family, and no phenotypic resistances were detected (7).

A colony of *E. coli* was inoculated in Lysogeny Broth (LB) (Merck, Milan, Italy), overnight cultured at 37°C. About 1 mL of the culture was aliquoted for DNA extraction using the Wizard Genomic DNA Purification Kit (Promega, USA). The strain was lysed, the RNA degraded, proteins separated, and DNA precipitated. Total DNA was quantified using Qubit dsDNA HS Assay Kit (Fisher Scientific, Italy). VAHTS Universal Plus DNA Kit was used for library preparation (Vazyme, China).

The genome was sequenced using the Illumina MiSeq v3 platform (BMR, Italy) generating 1,806,681 (2 × 300 bp) reads. After fastp (v.0.20.1) filtering, 95.7% of the remaining readings had an average Phred quality score of Q30 (8). SPAdes (v.3.6) was used for scaffold assembly (9). Contigs under 500 bp and below 2× coverage were discarded. Qualimap (v.2.2) showed a mean coverage of 182.59× (10). Assembly evaluation resulted in 40 high-quality contigs, with a total size of 4,712,575 bp and a final GC content of ~50.79%. Quast (v.5.0.0) revealed that the assembly *N*_50_ was 215,9 kb and the biggest contig measured 509,219 bp (11) Table 1. Quality was assessed with CheckM (v.1.0.18), which displayed a completeness of ~99.93% and 1,207 single-copy orthologous genes with no evidence of contaminant contigs (12). The sequenced strain appears to belong to the *E. coli* species, according to phylogenetic placement analysis carried out using GTDB-Tk (v.1.7.0) (13). Antibiotic resistance genes (ARGs) were searched with ABRicate (v1.0.0) (14). ARGs analysis showed the presence of beta-lactamase genes but the phenotypic resistance was under the EFSA cutoff value. By applying PROKKA (v.1.14.5), 4,664 genes have been identified, of which 4,357 were coding sequences (15). Neither plasmids (Platon v.1.16) nor genes belonging to the pks island for colibactin production were identified (16). Default parameters were used for all tools, except where otherwise noted.

**TABLE 1:** Genomic features of *E. coli* 5C LMG S-33222

Features	LMG S-33222
Contigs	86
Largest contig (bp)	509,219
Total length (Mb)	4.7
Coverage	182.6×
*N*_50_ (kb)	215.9
GC (%)	51

## Data Availability

The whole-genome shotgun project has been deposited in NCBI under the BioProject accession number PRJNA1114436 with the corresponding GenBank accession number JBDPNK000000000.1. Raw reads are deposited in the Sequence Read Archive under the accession number SRR29289048 and the assembly is available under the accession number GCA_039944155.1.
